# *In Silico* Predictions of Endocrine Disruptors Properties

**DOI:** 10.1210/en.2019-00382

**Published:** 2019-07-02

**Authors:** Melanie Schneider, Jean-Luc Pons, Gilles Labesse, William Bourguet

**Affiliations:** Centre de Biochimie Structurale, CNRS, INSERM, Université de Montpellier, Montpellier, France

## Abstract

Endocrine-disrupting chemicals (EDCs) are a broad class of molecules present in our environment that are suspected to cause adverse effects in the endocrine system by interfering with the synthesis, transport, degradation, or action of endogenous ligands. The characterization of the harmful interaction between environmental compounds and their potential cellular targets and the development of robust *in vivo*, *in vitro*, and *in silico* screening methods are important for assessment of the toxic potential of large numbers of chemicals. In this context, computer-aided technologies that will allow for activity prediction of endocrine disruptors and environmental risk assessments are being developed. These technologies must be able to cope with diverse data and connect chemistry at the atomic level with the biological activity at the cellular, organ, and organism levels. Quantitative structure–activity relationship methods became popular for toxicity issues. They correlate the chemical structure of compounds with biological activity through a number of molecular descriptors (*e.g.*, molecular weight and parameters to account for hydrophobicity, topology, or electronic properties). Chemical structure analysis is a first step; however, modeling intermolecular interactions and cellular behavior will also be essential. The increasing number of three-dimensional crystal structures of EDCs’ targets has provided a wealth of structural information that can be used to predict their interactions with EDCs using docking and scoring procedures. In the present review, we have described the various computer-assisted approaches that use ligands and targets properties to predict endocrine disruptor activities.

During the past decades, a large number of observations have shown that many exogenous substances can interfere with hormone levels or hormone action and, in turn, induce toxic effects. This has led to the identification of endocrine disrupting chemicals (EDCs) as a new class of toxic agents that will not be recognized, at first, by their chemical structure or by a specific type of usage but, rather, by their mechanisms of action (1–3). EDCs are exogenous substances that interfere with the function of hormonal systems and produce a range of developmental, reproductive, neurologic, immune, or metabolic diseases in humans and wildlife (4). Most EDCs are man-made chemicals produced by industry and released into the environment. However, some naturally occurring EDCs can also be found in plants or fungi. Exposure to EDCs occurs through ingesting food, drinking water, breathing contaminated air, or skin contact. The group of molecules acting as EDCs is highly heterogeneous and includes compounds that are often distantly related to endogenous ligands in terms of size or chemical structure. This group contains substances such as plasticizers (*e.g.*, bisphenols, phthalates), preservatives (*e.g.*, parabens), the byproducts of various industrial processes (*e.g.*, dioxins), surfactants (*e.g.*, alkylphenols, perfluoroalkyls), biocides (*e.g.*, organotins), flame retardants (*e.g.*, halogenated bisphenols), and ultraviolet filters (*e.g.*, benzophenones) and natural compounds such as the phytoestrogens genistein and daidzein or the mycoestrogen zearalenone.

EDCs can affect the endocrine systems of an organism in a wide variety of ways, for example, by mimicking natural hormones, antagonizing their action, or modifying their synthesis, metabolism, and transport through their interference with multiple cellular targets. These include membrane and nuclear receptors, the aryl hydrocarbon receptor, the enzymatic machineries involved in hormone biosynthesis and metabolism, and various carriers. Within the chemical regulations, criteria to identify EDCs have been recently proposed, which require information on a chemical’s endocrine mode of action and related adverse effects relevant for human health. This involves the screening and testing of EDCs and mainly incorporates internationally accepted test methods developed under the Organization for Economic Cooperation and Development. In this context, the development of accurate *in silico* testing strategies could help to elucidate or confirm the suspected mode of actions and might suggest associated adverse effects by predicting the repertoire of molecular targets of EDCs. It might also provide guidelines to select or optimize molecule usage or designed to prevent unwanted activities.

Approaches to predict toxicity or activity against a particular target for a putative EDC can be divided according to the nature of data they are using and by their demand in computational resources. One of the simplest tools is ADME (Absorption, Distribution, Metabolism, Excretion)-Tox filters often used by pharmaceutical companies. Those can be based on composition rules (5), for example, specific chemical groups that should be avoided because they have shown adverse effects in the past (6). Another method of investigating the problem is drug-induced metabolic perturbation studies. These are based on metabolic network modeling using large-scale “omics” data, metabolic stability estimations, and mode of action analyses (7–10). Some of them have been shared with usual ADME-Tox issues such metabolization by cytochromes P450. The general methods for *in silico* toxicity prediction have been previously reviewed (7, 11–13). EDCs fall into particular niches of the available chemical space optimized for other properties and only partially mimicking natural hormones. They often differ in chemical structure from most medicinal and endogenous compounds and are encountered at unexpectedly high concentrations in the environment and living organisms [*e.g.*, bisphenol A (BPA), organotins]. Therefore, dedicated approaches are needed to detect the endocrine disruption potential.

The focus of the present review was centered on the field of prediction methods that aim to qualify the interaction between given small molecules, as potential EDCs, and a focused set of macromolecular targets. This is a very large field of research with many different methods that have been developed. Each method has its strengths, limitations, scope of application, and specificity of interpretation. The first questions to be asked upfront include the following: How much data are available? What is the nature of this data? How fast are results required? What is the minimal required accuracy of the prediction? What resources are available? Having those questions in mind, the goal is to find the most effective method. In addition to the classification into high-, medium-, and low-throughput methods, the available approaches can be classified further according to the type of data used. Most often, chemoinformatics methods will be classified as ligand-based and target structure-based approaches (Fig. 1) (14). Depending on the amount of data and the need for screening large data sets, the corresponding method should be chosen. This clearly involves a tradeoff between the amount of molecules, speed, and accuracy. However, combinations of techniques are emerging to improve overall efficiency and applicability. We first surveyed ligand-based virtual screening techniques as quick filters and then the role of structure-based virtual screening and discussed their potential combination. In both cases, one must adequately describe the studied molecules, which will usually start by extracting or writing its chemical formula as a linear string of atoms, such as SMILES (simplified molecular-input line-entry system) (Fig. 2), to be subsequently transformed into various other representations [two-dimensional (2D), three-dimensional (3D)] either for comparison with other molecules (*i.e.*, similarity searches, properties comparisons) in ligand-based virtual screening or by docking into putative targets (*i.e.*, in structure-based virtual screening).

**Figure 1.**
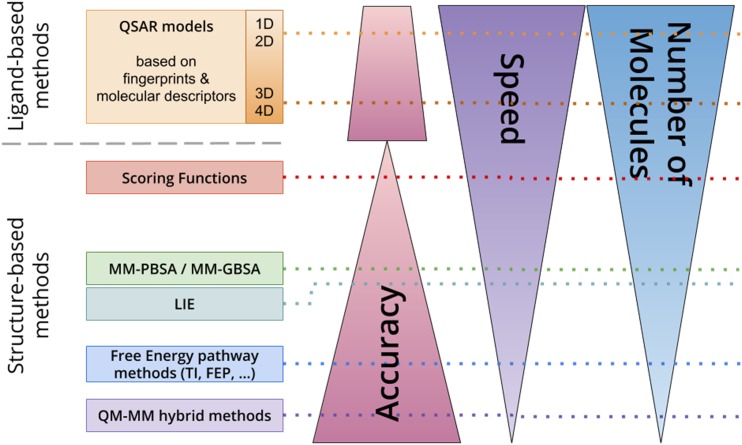
Affinity prediction methods grouped by ligand-based and structure-based methods and ranked by accuracy, computational effort and speed, and number of molecules that can be used. 1D, one-dimensional; 4D, four-dimensional.

**Figure 2.**
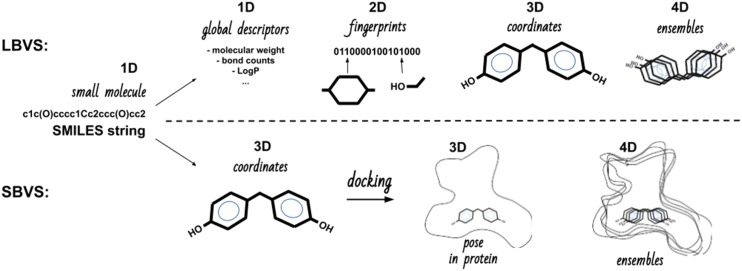
Molecular representations used by different methods, which were classified by the overall methodology (ligand-based virtual screening and structure-based virtual screening) and according to the dimensionality [one-dimensional (1D), 2D, 3D, four-dimensional (4D)] of the variables used.

## Ligand-Based Methods

The so-called quantitative structure activity relationship (QSAR)/quantitative structure property relationship prediction models have been developed to predict a particular activity or property of the molecule in question. The simplest approaches have been based on the calculation of molecular descriptors that consider the molecule as a whole entity and calculate one value for the whole molecule (*e.g.*, molecular weight). The least expensive in terms of computational cost are models based on binary representations of molecules, called molecular fingerprints, or molecular descriptors (Fig. 2). These fingerprint representations can be binary in nature (property present or absent, yes or no, or 1 or 0), which only reflects the presence (or not) of a given feature or a count representation (sum of the instances for each feature). Millions of compounds can be screened within a reasonable period. Different types of fingerprints represent different properties of molecules, and it is, therefore, crucial to select an adequate type for modeling the desired activity. Many different types of molecular descriptors are available and might already be an output of a property prediction. Molecular descriptors and chemical fingerprints can be classified according to their dimensionality (Fig. 2). One-dimensional descriptors are scalars that describe the molecule according to its chemical formula (*e.g.*, molecular weight, atom counts, or bond counts). Two-dimensional descriptors are based on the structural topology, such as fragment counts or functional group counts (*e.g.*, alcohol function or aromatic ring). Three-dimensional descriptors extract information from 3D coordinate representations and are, therefore, based on the molecule’s geometry. Four-dimensional descriptors are an extension of the 3D descriptors, which consider multiple conformations. In the case of 3D and four-dimensional descriptors, the computational effort will have already increased substantially and the borders toward the so-called structure-based methods will tend to vanish. All these descriptors allow for a rather rapid similarity search and classification to deduce or predict functional properties. Various *in silico* QSAR tools and, even, servers, namely the Organization for Economic Cooperation and Development QSAR toolbox (https://qsartoolbox.org/), VEGA HUB (https://www.vegahub.eu/), or CAESAR (http://www.caesar-project.eu/), to cite a few, are available, and open challenges have now been implemented to evaluate them more fairly, such as the Tox21 (“toxicity testing in the twenty-first century” initiative) project. DeepTox, the winner of the “Tox21 Data Challenge 2014” obtained excellent performances with a deep multitask neural network using ECFP4 fingerprint features (15).

In general, ligand-based methods will be very restricted to the chemical space of the molecules used for method development, especially if only a limited amount of data are available for model training. This can cause disappointing performances, especially in projections or extrapolation to new and dissimilar compounds (16). Therefore, the definition and declaration of an applicability domain—a region in the chemical space for which a QSAR model should make predictions with a given reliability—is considered as a necessary good practice for those model types (17). The quality of experimental data is also essential for valuable modeling as recently illustrated on the estrogen receptors (ER*α*, ER*β*), which are two of the most extensively studied targets with respect to endocrine disrupting effects (18, 19). Regulation rules have been devised by the US Food and Drug Administration that require the assessment of estrogenic activity, and effort have been made to predict for ER binding (20, 21), including a large collaborative project (22). The latter, which compared numerous models and data sets, showed that poorly evaluated data sets are of little help for improving prediction quality despite providing experimental data for thousands of ligands. Similarly, other steroid hormone receptors, such as the androgen receptor, have been targeted for model development (23–26). To evaluate the risk of being EDCs, the prediction of a specific mechanism such as binding to a particular receptor is preferred for its expected greater accuracy and low cost. General models that aim at predictions on large protein families are less common. EDCs are active against specific targets of diverse nature (enzymes such as cytochromes P450 or DNA-binding proteins such as nuclear receptors). Accordingly, dedicated models might be required in agreement with their experimental characterization.

## Structure-Based Methods

The increasing knowledge of functional and structural data has allowed for the evaluation or prediction of the potential interactions of known or putative EDCs to various targets using docking or more demanding approaches [*e.g.*, molecular dynamics (MD); see the next paragraph]. Structure-based methods, also called target-based methods, use information from a protein target 3D structure and are spanning a large scale in terms of computational cost. Docking procedures are the most widely used in virtual screening campaigns and can manage to thousands of ligands. They are based on sampling the conformational space of a given ligand in the binding pocket of a target molecule and a subsequent pose evaluation performed by scoring functions. Although the sampling of many widely used algorithms has seemed to be sufficient to find accurate poses (defined by reproducing crystallographic poses), the scoring functions still seem to suffer from diverse approximations (27–29). Accordingly, docking, followed by various rescoring procedures, is now commonly used to screen large molecular data sets in drug discovery (27, 29). This has been applied for endocrine disruption prediction on the androgen receptor (24, 25, 30) and other nuclear receptors (31–33). Automatic docking to 16 putative targets of EDCs or 14 distinct nuclear receptors has been made user-friendly through two servers, the OpenVirualToxLab (34) and Endocrine Disruptome (35). However, structure analysis has also revealed the importance of protein flexibility. Adequately modeling target flexibility is a major limitation that has been addressed using structure ensembles, instead of single conformations (36). One approach is to use multiple experimental conformations in parallel for docking and gather the results to extract the best or more likely poses. A derivative of our server for comparative modeling “@TOME” (37) now includes a docker (to be described in more detail elsewhere). This allows for the selection of the protein conformation best suitable to accommodate a given ligand. This dedicated server called EDMon (Endocrine Disruptor Monitoring; available at: http://edmon.cbs.cnrs.fr/) is now available to screen for ER*α*, ER*β*, and peroxisome proliferator–activated receptor-*γ* (PPAR*γ*). It predicts for affinities using a rescoring approach based on machine learning (38). However, the problem is still severe for promiscuous proteins, such as the nuclear receptors CAR (constitutive androstane receptor) and PXR (pregnane X receptor) (39). The dozen of structures described to date for these receptors have shown dramatic structural rearrangements on ligand binding, and more experimental 3D structures are necessary to reach a better description of the conformational landscape they could access.

Alternatively, to unravel or model intrinsic protein flexibility and possible ligand-induced fit, MD simulations can be used but at a significantly greater computational cost (*e.g.*, one to several weeks using a standard workstation). MD-based prediction methods require more effort with respect to system setup and analysis, and they are usually not provided as simple “plug and play” modules, such as is the case for many commercial or noncommercial docking tools. To date, MD simulations have already been used to study the structural flexibility and the dynamics of binding events of several nuclear receptors (40–45), with and without further investigation of small molecule-binding affinities. The server OpenVirualToxLab (34) provides easy access to focused MD, which is used to refine and evaluate theoretical complexes deduced from docking into 16 EDC targets. In general, MD-based affinity estimation protocols can be divided into two major groups: endpoint methods and free energy pathway methods. The endpoint methods, as already indicated by the name, consider the two “end” states of the system: the bound and the unbound molecules. Two commonly used ones include the MM-PBSA (molecular mechanics Poisson-Boltzmann surface area) (46, 47) and MM-GBSA (molecular mechanics generalized born surface area) (47–49). These computations can be adjusted to a particular system through parametrization within the so-called linear interaction energy method (24, 50–52). For example, MD simulations, followed by MM-PBSA calculations, have been used to study the structural effects and interaction mechanism of BPA with three human nuclear receptors, ER*α*, ERR*γ* (estrogen-related receptor-*γ*), and PPAR*γ* (53) or to determine the binding of bisphenols BPA, bisphenol AF, and bisphenol S to ER*α* (54). These computations require some expertise but can be performed using a personal workstation and are now often applied on several dozens of compounds against a given target. They allow for rescoring of docking poses using physics-based approaches; however, their usefulness has continued to be debated. Furthermore, the standard MD techniques can suffer from an insufficient sampling of the conformational space of the target molecule. This can occur for different reasons, such as large conformational movements during binding, slow transitions between states, rare events, or high-energy barriers that must be overcome. In such cases, a set of different computational methods has been proposed—the free energy pathway methods such as transition path sampling, umbrella sampling, steered-MD, and funnel-metadynamics (55–59). Among the free energy pathway methods is a subgroup of alchemical methods represented by the thermal integration (60, 61) and free energy perturbation (62, 63) methods. Recently, a combination of methods has been applied to toxicity studies for the identification of possible ligand binding modes to PPAR*γ* (64). However, those approaches are even more demanding in central processing unit time and are not commonly performed for toxicity predictions.

Finally, extremely precise energy estimations can be computed using quantum mechanics (QM) but at huge computational cost. Thus, QM is often restricted to modeling of the binding site. Mixed/hybrid approaches will allow for computation locally of a QM procedure, and a standard MD approach is applied to the rest of the molecular system under study. Quantum effects might be required to correctly estimate particular molecular interactions when atomic bonds are broken or reformed during the binding event or for predicting the reaction rates in drug metabolism, which is the case for cytochromes P450 (65–67). Free-energy estimation and QM have been performed on a very limited number of complexes. However, their exquisite characterization of molecular structures and interactions might help to precisely define various chemical properties (*i.e.*, conformation, charge, reactivity) and/or to parametrize quicker methods (*e.g.*, for scoring or docking).

## Current Limitations and Future Directions

Because the US ToxCast program and the European Union’s Registration, Evaluation, Authorization, and Restriction of Chemicals regulation aim to assess the toxicity of more than 100,000 synthetic chemicals, a strong demand exists for alternative test methods and, in particular, such computational tools that will allow for the reduction of the cost of the evaluation and in animal lives. Despite recent major advances in the field of affinity prediction resulting in numerous tools and diverse approaches, one must remember their limitations. One of the major concerns of ligand-based *in silico* prediction methods is its high dependency on experimental data. The presence of inconsistent and erroneous data during the training process can lead to biased and inaccurate predictions and the applicability domain is a major prerequisite that needs to fit for reliable predictions. Large-scale high-throughput experimental testing to generate coherent databases and curation of the existing ones would help generate more accurate prediction models. The QSAR approaches available usually display applicability domain centered on the training data and struggle to yield reasonable predictions for highly unbalanced data sets. The current development and combination of novel statistical and machine/deep learning approaches are likely to generate novel *in silico* models that could manage highly unbalanced data sets, allowing for the applicability domain to expand beyond the training data.

Concerning target-based methods, its dependency is more reduced. However, the issue of potentially unknown structural changes still exists. An inherent limitation of any modeling tool is its parametrization for all possible chemistries. Currently, knowledge is lacking regarding the proper evaluation of protein–ligand interactions involving halogen atoms, metals (*e.g.*, organotins), or newly used entities such as organoborans. In addition, such compounds have been previously demonstrated to act as EDCs. More crystal structures would be necessary to reflect the conformational landscape of the target receptors in a more comprehensive manner and help in training docking tools with exotic atoms. This suggests the need for tighter interactions between structuralists and predictors to tune experimental works to fill in the gaps in structural and/or functional data. Another difficulty not easily manageable, especially for large chemical data sets, is the possibility for simultaneous binding of two or more cases of the same molecule (especially for small compounds) and/or of distinct molecules (mixtures) in a cooperative and/or allosteric fashion. Developing dedicated tools will be necessary to manage this task correctly to predict potential “cocktail effects” (68). For different protein targets (16 listed to date for EDCs), different techniques are already available and have been applied with varying rates of success. Not only for nuclear receptors or cytochromes P450, but also for ion channels such as the *hERG* (human ether-a-go-go-related gene) potassium channel, different methods from ligand-based and target-based to systems biology have been applied (69).

Finally, cascading prediction tools and filters will be necessary to (i) account for potential metabolization that creates unexpected or new chemical entities with new properties, (ii) detect nonclassic properties [*e.g.*, covalent attachment (70), multiple binding], (iii) combine QSAR and docking, or (iv) start to predefine structural ensembles for quicker estimation of receptor flexibility to derive more accurate predictions. The latter might help in accessing better description of flexible complexes and avoid the burden of long simulations. Next, one could dream of combining those studies with mathematical models at the cellular level and in the endocrine system. Hence, room exists for further improvements in which a fruitful interplay between modeling and experimental characterization should be promoted.

## Abbreviations:

2D: two-dimensional
3D: three-dimensional
BPA: bisphenol A
EDC: endocrine-disrupting chemical
ER: estrogen receptor
MD: molecular dynamics
PPAR*γ*: peroxisome proliferator–activated receptor-*γ*
QM: quantum mechanics
